# Renal clearance rate, estimated renal reabsorption ratio, and kidney proteome of Holstein dairy cows with divergent milk urea concentrations

**DOI:** 10.3389/fmolb.2026.1819426

**Published:** 2026-10-27

**Authors:** Franziska Koch, Dirk Albrecht, Solvig Görs, Carolin B. M. Müller-Kiedrowski, Klaus Wimmers, Björn Kuhla

**Affiliations:** 1 Research Institute for Farm Animal Biology (FBN), Dummerstorf, Germany; 2 Department for Microbial Physiology and Molecular Biology, University of Greifswald, Greifswald, Germany; 3 Research and Teaching Extension for Alpine Ruminant Medicine, Clinical Center for Ruminants, Camelids and Herd Health Management, University of Veterinary Medicine Vienna (Vetmeduni), Vienna, Austria

**Keywords:** amino acids, carnosine, lactate, nitrogen excretion, proteomics, transporter, urea

## Abstract

Recent mRNA expression studies have proposed various renal proteins to be involved in the excretion of electrolytes, amino acids, and charged compounds to reduce urea, uric acid, and the creatinine clearance rates of dairy cows with high milk urea (HMU) concentrations. Hence, the aim of this study is to analyze the renal clearance of monovalent ions, amino acids and lactate, and the kidney proteome of dairy cows with high and low milk urea concentrations. HMU cows had higher plasma carnosine concentrations but lower renal carnosine clearance and transporter expression. HMU cows possessed increased plasma citrulline but reduced arginase abundance, suggesting downregulated urea detoxification. Pathway analysis revealed proteasomal activation to increase the degradation of proteins which could clog the filter barrier in HMU cows. However, reduced urea, uric acid, and the creatinine clearance rates of HMU cows could not be explained by reduced expression of their transporters but by altered local solute, such as carnosine handling and a lighter relative kidney weight.

## Graphical Abstract

**Figure:**
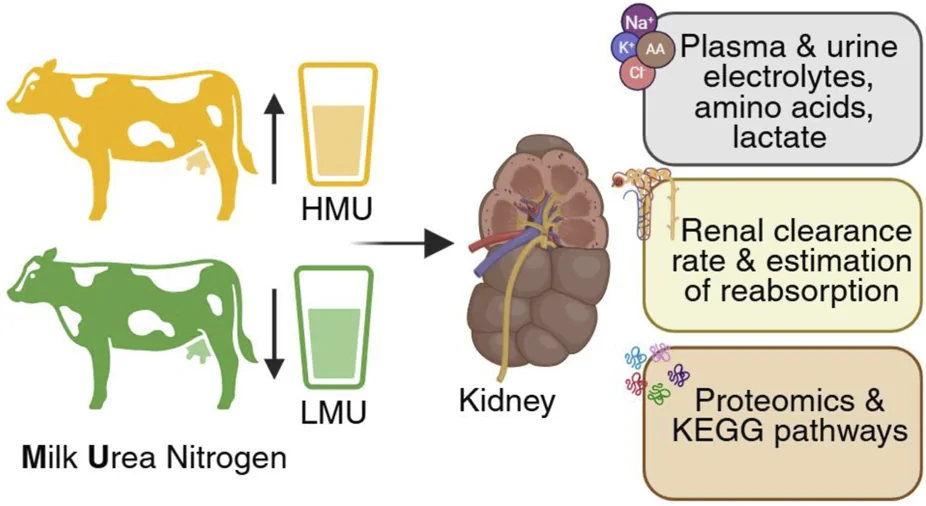

## 1 Introduction

Livestock is responsible for 23% of global nitrous oxide and 60% of global ammonia emissions, which negatively impact soil, water, and air quality and which ultimately alter biodiversity (FAO, 2025; Liu et al., 2022). The major source contributing to these nitrogen emissions is manure originating from stables or pasture farming, where faecal-derived urease rapidly converts urinary urea into ammonia (Nu et al., 2022). The level of urinary urea excretion depends on the amount of ingested crude protein (CP), which, when ruminally degraded, yields ammonia and ammonium ions, which are both neutralized in the liver to form urea (Abdoun et al., 2006). This is then excreted via urine but also via faeces and milk (Abdoun et al., 2006). Because the secretion of milk urea nitrogen (MUN) is proportional to the dietary CP concentration, MUN concentration serves as a suitable indicator to monitor optimal CP content in the diet, at least at a herd level (Burgos et al., 2007; Mutsvangwa et al., 2016; Aguilar et al., 2012). However, individual dairy cows may differ in terms of their MUN concentration, despite consuming the same amount of dietary CP; low MUN concentrations have thus been proposed as a suitable predictor of diminished urinary nitrogen excretions in breeding programs (Marshall et al., 2020; Jahnel et al., 2023). Previous studies have shown that dairy cows with high milk urea nitrogen (HMU) have lower or tend to have lower renal clearance rates for urea, creatine, creatinine, and uric acid, as well as lower kidney weights than cows with low milk urea nitrogen (LMU) (Muller et al., 2021; Prahl et al., 2023). However, total urinary urea and nitrogen excretions were similar between cows possessing average HMU or LMU (Muller et al., 2021; Prahl et al., 2023). Therefore, LMU concentrations seem not necessarily linked to lower urinary nitrogen excretions or a lower nitrogen footprint. However, more research is required to elucidate the physiological and molecular mechanisms underlying the different renal clearance rates between HMU and LMU cows.

The kidney plays a key role in the excretion of various water-soluble nitrogen metabolites from blood circulation, thereby regulating respective plasma metabolite concentrations. The filtration of urea, ammonia, and creatinine predominantly takes place in the renal cortex before they are reabsorbed in the proximal and distal tubules. Urea transport is facilitated primarily by the urea transporter A (*SLC14A2*) and aquaporins (e.g., *APQ3*, *AQP7*, and *AQP10*) (Li and Wang, 2014), whereas creatinine transport is facilitated via the solute carrier family 6 member A8 (*SLC6A8*) (Snow and Murphy, 2001) and ammonia via the Rh family C glycoprotein (*RHCG*) (Weiner and Verlander, 2014). The filtration of uric acid is facilitated via *SLC22A12*, but in the bovine kidney, uric acid is also synthesized from xanthine via xanthine dehydrogenase (*XDH*), thereby adding to total urinary uric acid excretion. Interestingly, targeted mRNA expression analysis reveals a tendency to a higher abundance of *APQ3* in the renal cortex of HMU compared to LMU cows, highlighting one potential reason for the more effective urea reabsorption of HMU animals (Reyer et al., 2024). In addition, Reyer et al. (2024) reported a higher mRNA expression of the neutral amino acid transporter *SLC6A19* and a lower expression of the proline transporter *SLC6A7* and the neutral amino acid transporter *SLC38A2* in the kidney of HMU compared to LMU cows. The excretion of free amino acids (FAA) via the kidneys contributes to total urinary nitrogen excretion, but the renal amino acid clearance rates in cows differing in MUN have not been investigated.

The kidney further controls electrolyte homeostasis, and sodium (Na^+^), chloride (Cl^−^), and potassium (K^+^) play a key role in regulating water secretion and reabsorption. About two-thirds of Na^+^ ions are actively reabsorbed by Na^+^/K^+^ ATPase, generating a gradient that allows other electrolytes, such as Cl^−^, to follow passively via diffusion (solvent drag-like transport) (Gunzel, 2023). The plasma concentration of monovalent ions affects their excretions with urine, and the osmotic gradient in the tubules produced by these ions may influence the renal excretion and reabsorption of charged N metabolites. It has been demonstrated that increased Na^+^ intake negatively affects MUN concentrations and urine volume but not urinary nitrogen excretions of dairy cows (Spek et al., 2012a; Spek et al., 2013). Whether renal proteins involved in the excretion of electrolytes, FAA, or other charged compounds contribute to the divergent urea, uric acid, and creatinine clearance rate of HMU and LMU dairy cows remains to be elucidated. Therefore, the objective of the present study was to analyze and compare the renal clearance rate and renal reabsorption ratio for Na^+^, Cl^−^, FAA, and lactate and to analyze the proteome of the kidney of Holstein dairy cows with divergent MUN.

## 2 Materials and methods

### 2.1 Animals and feeding

The experimental procedure was approved by the state office of Agriculture, Food Security and Fishery Mecklenburg-Western Pomerania, Rostock, Germany (LALLF 7221.3–1-052/17) as described by Muller et al. (2021) and Prahl et al. (2022). All experiments and methods were performed in accordance with relevant guidelines and regulations following the ARRIVE guidelines (Percie du Sert et al., 2020). In brief, 18 non-pregnant, multiparous German Holstein cows were screened for milk yield and high and low MUN from the last five monthly milk testing of commercial farms in Mecklenburg-Western Pomerania. Each pair, comprising one LMU and one HMU cow, entered the experiment between September 2018 and February 2020 in nine blocks. Farmers were informed and agreed to the use of cows in the present study. Animals were in second to fourth lactation with comparable milk yield (HMU: 32.9 ± 1.0 kg/d, n = 9; LMU: 31.5 ± 1.4 kg/d; n = 9), high (HMU: 285.9 ± 11.5 mg/L) and low (LMU: 190.5 ± 33.7 mg/L) MUN, and a body condition score (BCS) of 3.4 ± 0.1 on a five-point scale. The HMU–LMU pairs were bought and transported to the experimental free-range barn of the Research Institute for Farm Animal Biology (FBN). The cows were fed twice daily, at 0500 h and 1700 h, a total mixed ration (crude protein: 15.0% ± 0.3%; metabolizable energy: 10.9 ± 0.2 MJ/kg, each on the dry matter (DM) basis; Table 1) for 2 weeks *ad libitum* and milked at 0430 h and 1630 h. After 2 weeks of adaptation to the diet, the cows were transferred from the barn to a climate-controlled room at 15 °C and adapted to a tie-stall on a 2.4 × 1.6 m stanchion for 1 week. They were equipped with a jugular catheter (Cavafix Certo m. Splittocan, 32 cm length; B. Braun Melsungen AG, Melsungen, Germany) and a urinal consisting of a customized synthetic leather model with a cut-out around the vulva area for 4 days (Muller et al., 2021; Prahl et al., 2022). On day 3 of the sampling period, blood was sampled after the morning feeding in K_3_-EDTA-containing tubes and centrifuged at 1,345 × g for 20 min at 4 °C. The plasma aliquots thus obtained were stored at −20 °C until further analysis. Urine was collected daily in a container containing 200 mL 30% H_2_SO_4_. Urine volume and weight were determined, and acidified urine aliquots were taken and stored at −20 °C until further analysis. On day 5, the cows were transferred to the institute’s slaughterhouse, weighed, anesthetized by captive bolt stunning, and exsanguinated for tissue collection. Their kidneys were weighed, and samples from the renal cortex and medulla were immediately chilled on ice, cut into smaller pieces, shock-frozen in liquid nitrogen, and stored at −80 °C until further analysis.

**TABLE 1:** Feed constituents, nutrient composition, and energy concentration of the total mixed ration (TMR; means ± SEM).

Ingredient (g/kg of DM)	TMR
Grass silage	227.4 ± 19.0
Corn silage	368.7 ± 15.5
Forage rye silage	82.5 ± 35.9
Barley straw	4.2 ± 4.2
Corn meal	70.8 ± 14.8
Wheat seeds	131.2 ± 8.9
Rapeseed extraction meal	99.6 ± 10.6
Mineral feed^1^	10.3 ± 0.4
Limestone^2^	3.7 ± 0.3
Feed salt^3^	1.6 ± 0.0
Nutrients (g/kg of DM)	
Crude ash^4^	75.3 ± 9.3
Crude fat	29.5 ± 1.2
Crude protein	150.3 ± 3.3
Crude fiber	179.1 ± 4.2
ADF	207.8 ± 4.2
NDF	388.4 ± 8.0
Starch	265.1 ± 7.9
Sugar	14.5 ± 2.3
ME, MJ/kg DM	10.9 ± 0.2
Utilizable crude protein^5^	131.8 ± 14.9

1 Panto Mineral R 8609 (HL, Hamburger Leistungsfutter GmbH, Hamburg, Germany), composition: 20% calcium, 6% phosphorous, 8% sodium, 6% magnesium, 0.03% inorganic nitrogen, 13.74% phosphorous pentoxide. Additives per kg original substance: 900,000 IU vitamin A; 200,000 IU vitamin D3; 4.5 g vitamin E; 1.5 g Cu; 8 g Zn; 5 g Mn; 60 mg I; 21 mg Co; 50 mg Se.

2 Bergophor CaCO3 V001 (Hohburg Mineralfutter GmbH, Lossatal, Germany): 37% calcium.

3 Animal feed salt (ESCO—European Salt Company GmbH and Co.KG, Hanover, Germany): 38% sodium, 0.3% calcium, 0.01% magnesium.

4 Measured quantity elements g/kg: calcium 6.2 ± 0.8, phosphorous 3.7 ± 0.5, sodium 2.1 ± 0.4, magnesium 2.1 ± 0.2, potassium 11.5 ± 2.9.

5 Utilizable crude protein (g/kg DM) = [11.93 – (6.82 × UDP) (g/kg DM)/crude protein (g/kg DM)] × ME (MJ/kg DM) + 1.03 × UDP (g/kg DM), with UDP, undegradable protein (GfE, 2001). DM, dry matter.

### 2.2 Electrolyte analysis in plasma and urine

Urine samples collected daily were thawed on ice and proportionally pooled according to daily urine excretion before analysis. Pooled urine and plasma samples were diluted with ultrapure water 1:2.5 for urinary Cl^−^, 1:20 for urinary K^+^, and all others were undiluted and measured on a Fuji Dri-Chem 4000i (FUJIFILM, Tokyo, Japan). Plasma K^+^ could not be measured due to the utilization of K_3_-EDTA as preservative.

### 2.3 Creatinine and lactate analysis in plasma and urine

Plasma creatinine and lactate concentrations were analyzed photometrically using an ABX Pentra C400 Clinical Chemistry Analyzer (HORIBA Europe GmbH, Oberursel, Germany) using A11A01-907 (creatinine) and −721 (lactate; HORIBA ABX SAS, Montpellier, France). Urinary creatinine and lactate concentrations were determined in pooled urine samples by high-performance liquid chromatography (HPLC) (Muller et al., 2021; Metges et al., 2014).

### 2.4 Amino acid analysis in plasma and urine

Plasma and urinary FAA concentrations were analyzed by HPLC (1260 Infinity II system, Agilent Waldbronn, Germany) (Kuhla et al., 2010; Sciascia et al., 2025). In brief, samples were diluted 1:10 with ultrapure water. Free amino acids in pooled urine and plasma samples were separated after pre-column derivatisation with ortho-phthalaldehyde/3-mercaptopropionic acid (primary amino acids) and 9-fluorenylmethoxycarbonyl chloride (secondary amino acids) and prior blocking of SH groups with iodoacetic acid after the cleavage of sulfur bridges with 3-mercaptopropionic acid using a 250 × 4.6 mm Gemini® 5 μm C18 110 Å HPLC column with a 4 × 3 mm pre-column (both Phenomenex, Aschaffenburg, Germany) and a fluorescence detector. Chromatographic separation was performed at 40 °C using a gradient of 40 mM phosphate buffer (pH 7.45) and acetonitrile/methanol/water (45:45:10) ranging from 7% to 100% at a flow rate of 0.8 mL/min for 48 min. Ortho-phthalaldehyde derivatives were detected at 340 nm excitation and 450 nm emission and the 9-fluorenylmethoxycarbonyl derivatives at 266 nm excitation and 305 nm emission. All FAA and their metabolites were quantified against multipoint calibrated standards. Urinary glycine was ignored in the analysis due to the acidification of the samples causing hippuric acid cleavage into glycine and benzoic acid. Free isoleucine was not detectable in tenfold diluted urine.

The renal clearance rate and estimated reabsorption ratio were determined. The renal clearance rate for free amino acids (RCR_FAA_) was calculated as follows:

$${RCR}_{FAA}\left[L/\min\right]=\frac{\left(\frac{urinaryFAAexcretion\left[\frac{mmol}{d}\right]}{plasmaFAAconcentration\left[\frac{mmol}{L}\right]}\right)}{1140\left[\frac{\min}{d}\right]}.$$

The estimated renal reabsorption ratio for FAA (RRR_FAA_) relative to the renal creatinine clearance rate (RCR_creatinine_) was calculated as follows (Muller et al., 2021):

$${RRR}_{FAA}=1-\frac{{RCR}_{FAA}}{{RCR}_{creatine}}.$$

The RCR and RRR for Cl^−^, Na^+^, K^+^, and lactate were calculated accordingly.

### 2.5 Proteome analysis

Kidney tissues from the cortex and medulla (equal proportions) were ground with mortar and pestle under liquid nitrogen. For protein extraction, 50 mg tissue powder and 200 µL lysis buffer consisting of Tris-HCl (50 mM; Carl Roth), EDTA (1 mM; GE Healthcare), NaF (10 mM: Thermo Fisher Scientific), IGEPAL CA-630 (1% v/v; Sigma-Aldrich), Triton X-100 (1% v/v), sodium deoxycholate (DOC; 0.5% v/v), sodium dodecyl sulfate (SDS; 0.1% w/v), and a Roche complete Protease Inhibitor Cocktail tablet (one tablet per 10 mL buffer; Roche Diagnostic, Mannheim, Germany) were homogenized for 45 s. Samples were centrifuged at 4 °C and 16,100 × g for 10 min. Protein concentration was measured via the Bradford method and bovine serum albumin as standard. Protein extracts (25 µg per lane) were run on a 15% SDS-PAGE (Supplementary Figure 1). The resultant gel was stained with Coomassie Brilliant Blue (Serva Electrophoresis GmbH, Heidelberg, Germany) overnight and washed with distilled water. One lane per animal was cut into ten slices (180 slices in total; Supplementary Figure 2) and subjected to HPLC and mass spectrometry analysis. Each slice was transferred into a 1.5 mL reaction tube and washed twice with a 100 µL solution of 50% CH_3_OH and 50% 50 mM NH_4_HCO_3_ for 30 min and once with 100 µL of 75% CH_3_CN for 10 min. Samples were dried at 37 °C for 20 min and incubated with 4 μg/mL trypsin solution overnight at 37 °C. For extraction, gel slices were covered with 60 µL of 0.1% trifluoroacetic acid in 50% CH_3_CN and incubated under shaking for 30 min. The peptide containing supernatant was transferred into a clear glass vial and dried at 45 °C for 100 min in a concentrator (Eppendorf, Hamburg, Germany). The dry peptides (non-reduced or alkylated) were resuspended in 10 µL of CH_3_CN/H_2_O/trifluoroacetic acid (50%/49.5%/0.5%). They were analyzed using a Thermo Scientific ™ Q Exactive HF Orbitrap LC-MS/MS System with a LC: Thermo ™ Scientific EASY-nLC™ 1,200 (Thermo Scientific, Waltham, MA, United States). The peptides were separated using a custom capillary column: Postnova quartz glass capillaries, 20 cm long with 100 µm column inner diameter + ReproSil-Pur 120 C18-AQ, 3 µm (Dr.Maisch GmbH, Ammerbuch-Entringen, Germany). A gradient of 0.73%/min (eluent A = 0.1% (v/v) acetic acid in water LC-MS grade; eluent B = 80% acetonitrile with 0.1% acetic acid LC-MS grade at a flow rate of 0.3 300 nL/min was applied. For MS and MS/MS analysis, a complete overview scan was performed in Q Exactive HF with a mass range of 333–1,650 m/z and Fourier transform (FT) resolution of 60,000. Data acquisition was performed in data-dependent data acquisition (DDA) mode. Fragmentation of the 15 most intense precursor ions (TopN = 15), isolation window 1.4 m/z, was performed with the following settings: HCD (high-energy collision dissociation), normalized collision energy (NCE): 27; measurement of fragment ions in Orbitrap, resolution 15,000 at 200 m/z. In further settings, ions with the following charge states were excluded from the MS/MS analysis: unassigned charge state, charge 1, 5–8 or higher; dynamic exclusion time, 30 s (to prevent multiple fragmentation of the same precursor ion). The mass spectrometry was deposited to the ProteomeXchange Consortium via the PRIDE partner repository (Perez-Riverol et al., 2019). Data files were searched against the National Center for Biotechnology information bovine database (http://www.ncbi.nlm.nih.gov/) using Mascot version 2.6.2 with the common contaminant “Keratin” specified. The Mascot search was carried out considering the following parameters: parent ion mass tolerance of 10 ppm, fragment ion mass tolerance of 0.80 Da, and Met oxidation (+ 15.99492 Da).

Each Mascot search included the data from all ten gel slices per lane, and results were loaded into Scaffold software (version 5.0.1., Proteome Software Inc., Portland, OR, USA). The Scaffold viewer was utilized to validate MS/MS based peptide and protein identifications. Peptide identifications were accepted with two peptides uniquely characterizing one protein with 95% probability to achieve a false discovery rate less than 0.1% by the Peptide Prophet algorithm with Scaffold delta-mass correction and a false discovery rate of 3.5% (Supplementary Table 1). Only proteins identified in at least seven of nine animals per group were considered for further analysis. For the analysis of differential protein expression, raw spectral counts were processed utilizing the DESeq2 package of the bioconductor repository in R (www.bioconductor.org). The raw spectral counts and the metadata were used to generate a DESeqDataSet object with DESeqDataSetFromMatrix (Supplementary Table 2). The DESeq function performed estimate size factors, estimate dispersions, and negative binomial WALD test analysis with p-value criteria of 0.05. The sparse partial least squares-discriminant analysis (sPLS-DA) of 4,463 proteins was generated within the DESeq package. Volcano plotting and heat maps were utilized to visualize statistical differences with the R packages EnhancedVolcano (EnhancedVolcano: Publication, 2018) and ComplexHeatmap (Supplementary Figure 2) (Gu, 2022). Differentially regulated proteins were analyzed for divergent MUN and subjected to functional enrichment analysis using the Database for Annotation, Visualization and Integrated Discovery (DAVID, v2023q4, with updates from August 2025) (Huang da et al., 2009). The unique list of differentially expressed proteins with official gene symbols were submitted as gene list and the *Bos taurus* database as background. The cut-off value of the Benjamin-Hochberg factor was 0.05. Only the results from Gene Ontology (GO) and Kyoto Encyclopedia of Genes and Genomes (KEGG) pathway analyses were selected for functional annotation categories (https://davidbioinformatics.nih.gov/summary.jsp). KEGG pathways related to human diseases were excluded (Supplementary Table 3). The fold enrichment was calculated based on the ratio of two proportions:

$$Fold\;enrichment=\frac{\frac{m}{n}}{\frac{M}{N}},$$

where *m* is the number of target items in the gene hit list, *n* is the total number of genes in the list, *M* is the number of genes in the background list, and *N* is the total number of background genes.

### 2.6 Statistical analysis

The data were analyzed with measurement analyses of variance using the MIXED SAS procedure (version 9.4; SAS Institute Inc., Cary, NC, USA). The ANOVA model included the fixed factors MUN (levels: HMU/LMU), block (levels: 1-9), and days in milk (DIM) as covariables. Least-square means (LSmean) and their standard error of the mean (SEM) were computed for each fixed effect. The pairwise differences were tested using the Tukey–Kramer procedure. Results with *p* < 0.05 were considered significant and 0.05<*p* <0.1 as a trend.

## 3 Results

### 3.1 Animal characteristic and milk composition

Cow groups with divergent MUN did not differ in body weight, dry matter and water intake, milk yield, milk protein, milk fat, milk lactose, somatic cell count, ECM, and fat–protein quotient (Table 2). Per the design, the LMU group had lower milk urea concentration than the HMU group (*p* < 0.05), and milk urea secretion per day tended to be lower for LMU than HMU cows (*p* = 0.09). The percentage of the right kidney weight (*p* < 0.05) and the total kidney weight (*p* < 0.09), each relative to the body weight, tended to be higher in LMU than in HMU cows.

**TABLE 2:** Intake, performance, and weight of dairy cows with high (HMU, n = 9) and low milk urea concentration (LMU, n = 9). Data given as LSmeans ± SEM.

Parameter	HMU	LMU	SEM	*p*-value
Dry matter intake (kg/d)	17.0	17.1	0.7	0.932
Water intake (L/d)	57.8	64.4	4.8	0.340
Milk yield (kg)	22.8	23.4	1.6	0.770
Milk protein (%)	3.64	3.65	0.11	0.974
Milk fat (%)	4.43	4.22	0.21	0.461
Milk lactose (%)	4.73	4.76	0.06	0.702
SCC (x10^3^ cells/mL)	650.8	427.1	397.1	0.696
Milk urea concentration (mg/L)	262.9	200.2	16.0	0.015
Milk urea yield (mg/d)	5,806.7	4,528.0	512.2	0.09
ECM (kg/d)^1^	24.3	24.3	1.7	0.991
Fat–protein quotient	1.22	1.16	0.05	0.318
Body weight at slaughter (kg)	673.5	704.6	22.1	0.338
Percentage total kidney weight rel. to body weight (%)	0.25	0.27	0.008	0.094
Percentage kidney weight right rel. to body weight (%)	0.13	0.14	0.004	0.042
Percentage kidney weight left rel. to body weight (%)	0.13	0.13	0.005	0.324

ECM, energy corrected milk; SCC, somatic cell count.

1 ECM = [0.038 x fat (g) + 0.024 × protein (g) + 0.017 × lactose (g)] × milk (kg/d)/3.14.

### 3.2 Electrolyte, lactate, creatinine, and amino acid concentrations

There were no differences in plasma Na^+^, Cl^−^, lactate, and creatinine concentrations between LMU and HMU cows (Table 3). Furthermore, none of the proteinogenic or total amino acid concentrations differed between phenotypes, but citrulline and carnosine concentrations were significantly higher in the HMU than LMU group (*p* < 0.05, respectively; Table 3).

**TABLE 3:** Plasma electrolyte, lactate, creatinine, and free amino acid concentrations of dairy cows with high (HMU, n = 9) and low (LMU, n = 9) milk urea concentration. Data given as LSmeans ± SEM.

Parameter (mmol/L)	HMU	LMU	SEM	*p*-value
Chloride	97.8	94.0	2.7	0.328
Sodium	138.4	134.1	3.0	0.315
Lactate	4.7	5.1	0.4	0.487
Creatinine	98.1	92.7	3.0	0.234
Parameter (µmol/L)				
Aspartate	4.2	4.5	0.5	0.651
Glutamate	69.4	37.3	5.7	0.288
Cysteine	44.1	43.0	3.4	0.695
Alpha-aminoadipic acid	4.6	4.0	0.5	0.416
Asparagine	39.5	43.0	4.5	0.590
Serine	74.9	79.6	4.7	0.491
Glutamine	273.3	286.1	15.7	0.572
Histidine	38.5	37.7	5.6	0.925
Glycine	289.5	322.6	15.3	0.149
Threonine	91.0	88.9	8.9	0.869
Citrulline	89.8	68.1	5.5	0.015
Arginine	76.3	78.7	7.1	0.817
Pi-methylhistidine	5.3	5.0	0.6	0.734
Tau-methylhistidine	5.6	4.9	0.5	0.426
Pi + tau-methylhistidine	10.8	9.9	1.0	0.512
Beta-alanine	2.2	2.8	0.3	0.188
Carnosine	19.5	13.2	1.3	0.003
Alanine	269.2	262.5	13.8	0.736
Taurine	46.8	47.5	4.4	0.913
Tyrosine	40.6	38.5	3.4	0.669
Alpha-aminobutyric acid	22.8	19.6	1.9	0.252
Valine	215.7	197.2	16.7	0.445
Methionine	20.7	21.0	1.9	0.900
Tryptophane	53.0	50.6	3.9	0.668
Phenylalanine	48.6	44.9	2.5	0.313
Isoleucine	111.1	103.7	7.0	0.464
Ornithine	35.7	34.4	4.0	0.840
Leucine	116.1	104.8	8.9	0.379
Lysine	66.5	63.4	6.7	0.826
Proline	84.8	93.1	7.6	0.454
Sum of AA	2,267.4	2,255.2	101.2	0.933

Giving that isoleucine was not detectable in urine samples, we found no urinary amino acid concentration to differ between cow groups, except glutamate concentration, which tended to be lower in HMU than LMU cows (*p* = 0.095; Table 4). In addition, urine pH and volume and urinary Na^+^, K^+^, Cl^−^, and lactate concentrations were comparable between LMU and HMU cows (Table 4).

**TABLE 4:** Urinary pH, electrolyte, lactate, creatinine, and free amino acid concentrations, and 24-h urine excretion of dairy cows with high (HMU, n = 9) and low (LMU, n = 9) milk urea concentration. Data given as LSmeans ± SEM.

Parameter	HMU	LMU	SEM	*p*-value
Urine volume (L)	14.3	14.6	0.9	0.798
pH	8.8	8.7	0.1	0.928
Parameter (mmol/L)				
Chloride	246.6	256.0	18.3	0.827
Sodium	116.4	122.6	6.7	0.530
Potassium	219.1	212.0	9.2	0.592
Lactate	1.4	0.9	0.3	0.308
Creatinine	8.1	7.4	0.6	0.441
Parameter (µmol/L)				
Aspartate	46.8	53.3	6.0	0.288
Glutamate	129.5	175.1	18.1	0.095
Cysteine	155.6	158.3	16.8	0.910
Alpha-aminoadipic acid	97.3	96.2	12.1	0.947
Asparagine	16.7	16.7	1.0	0.327
Serine	60.9	57.7	10.8	0.838
Histidine	22.9	22.3	5.3	0.937
Glutamine	90.8	94.8	15.9	0.861
Threonine	55.3	49.0	14.2	0.761
Citrulline	5.8	5.6	1.8	0.920
Arginine	10.4	13.5	1.8	0.265
Pi-methylhistidine	27.8	27.9	3.0	0.967
Tau-methylhistidine	98.7	98.2	8.3	0.964
pi + tau-methylhistidine	126.5	126.1	10.8	0.982
Beta-alanine	127.5	154.2	27.3	0.500
Carnosine	33.9	34.6	4.2	0.918
Iso-aminobutyric acid	40.1	43.4	2.8	0.395
Alanine	437.7	547.5	134.3	0.573
Taurine	566.6	648.7	159.2	0.721
Tyrosine	11.0	10.7	2.3	0.908
Alpha-aminobutyric acid	0.79	0.81	0.12	0.913
Valine	7.9	7.0	2.4	0.800
Methionine	6.8	5.4	3.8	0.781
Tryptophane	20.4	18.7	3.6	0.745
Phenylalanine	3.2	3.5	1.1	0.862
Leucine	0.8	1.3	0.3	0.216
Ornithine	26.5	26.4	3.5	0.985
Lysine	44.8	43.6	4.3	0.846
Proline	11.1	12.3	1.7	0.624

### 3.3 Renal clearance rates and reabsorption ratios

We found no significant differences in the clearance rates of any electrolyte (Na^+^, and Cl^−^) or amino acid between groups (*p* > 0.1); however, the renal clearance rate for carnosine tended to be lower in HMU than LMU cows (*p* = 0.095; Table 5). In addition, the renal reabsorption ratio for electrolytes, creatinine, lactate, and FAA did not differ between cow groups (*p* > 0.1; Table 6).

**TABLE 5:** Renal clearance rates for electrolytes, lactate, creatinine, and free amino acids in dairy cows with high (HMU, n = 9) and low (LMU, n = 9) milk urea concentration. Data given as LSmeans ± SEM.

Renal clearance rate (L/min)	HMU	LMU	SEM	*p*-value
Chloride	0.025	0.028	0.002	0.431
Sodium	0.008	0.009	0.001	0.378
Lactate	0.003	0.003	0.001	0.303
Creatinine	0.801	0.863	0.052	0.474
Aspartate	0.126	0.143	0.023	0.599
Glutamate	0.019	0.023	0.003	0.278
Cysteine	0.036	0.036	0.004	0.923
Alpha-aminoadipic acid	0.218	0.242	0.035	0.647
Asparagine	0.004	0.005	0.001	0.430
Serine	0.008	0.007	0.001	0.714
Histidine	0.006	0.006	0.001	0.901
Glutamine	0.003	0.004	0.001	0.935
Threonine	0.007	0.006	0.002	0.729
Citrulline	0.001	0.001	<0.000	0.629
Arginine	0.001	0.002	<0.000	0.272
Pi-methyl-histidine	0.056	0.058	0.007	0.811
Tau-methyl-histidine	0.193	0.216	0.030	0.610
Pi-tau-methyl-histidine	0.124	0.133	0.017	0.714
Beta-alanine	0.920	0.642	0.196	0.333
Carnosine	0.018	0.028	0.004	0.095
Alanine	0.016	0.021	0.005	0.469
Taurine	0.109	0.137	0.033	0.575
Tyrosine	0.003	0.003	0.001	0.941
Alpha-aminobutyric acid	0.0004	0.0005	<0.000	0.401
Valine	0.0003	0.0003	<0.000	0.886
Methionine	0.003	0.003	0.001	0.857
Tryptophane	0.004	0.004	0.001	0.953
Phenylalanine	0.0006	0.0007	<0.000	0.857
Leucine	0.0001	0.0001	<0.000	0.129
Ornithine	0.007	0.009	0.001	0.240
Lysine	0.007	0.008	0.001	0.705
Proline	0.001	0.001	<0.000	0.904

**TABLE 6:** Estimated renal reabsorption ratio of electrolytes, lactate, and amino acids in dairy cows with high (HMU, n = 9) and low (LMU, n = 9) milk urea concentration. Data given as LSmeans ± SEM.

Estimated renal reabsorption ratio	HMU	LMU	SEM	p-value
Chloride	0.965	0.967	0.005	0.741
Sodium	0.989	0.989	0.002	0.867
Lactate	0.99	0.99	0.00	0.348
Aspartate	0.84	0.84	0.03	0.954
Glutamate	0.78	0.97	0.00	0.486
Cysteine	0.95	0.96	0.00	0.509
Alpha-aminoadipic acid	0.73	0.72	0.04	0.896
Asparagine	0.99	0.99	0.00	0.883
Serine	0.99	0.99	0.00	0.527
Histidine	0.99	0.99	0.00	0.631
Glutamine	1.00	1.00	0.00	0.818
Threonine	0.99	0.99	0.00	0.553
Citrulline	0.99	1.00	0.00	0.738
Arginine	1.00	1.00	0.00	0.370
Pi-methylhistidine	0.93	0.93	0.01	0.953
Tau-methylhistidine	0.76	0.75	0.04	0.805
Pi + tau-methylhistidine	0.84	0.84	0.02	0.965
Carnosine	0.98	0.97	0.01	0.228
Alanine	0.98	0.98	0.01	0.545
Taurine	0.85	0.84	0.05	0.822
Tyrosine	1.00	1.00	0.00	0.640
Alpha-aminobutyric acid	1.00	1.00	0.00	0.531
Valine	1.00	1.00	0.00	0.718
Methionine	1.00	1.00	0.00	0.589
Tryptophane	1.00	1.00	0.00	0.677
Phenylalanine	1.00	1.00	0.00	0.956
Leucine	1.00	1.00	0.00	0.154
Ornithine	0.99	0.98	0.01	0.565
Lysine	0.99	0.98	0.00	0.886
Proline	1.00	1.00	0.00	0.815

### 3.4 Functional classification of differentially expressed proteins

As a result of the renal cortex and medulla proteome analysis, we identified a total of 6,793 proteins, of which 4,463 remained after cleaning the dataset (Supplementary Table 1). The result of sPLS-DA revealed a discrimination between the protein expression of each group and explained 7% of the variation by component 1 and 5% of the variation by component 2 (Figure 1A). The loading components for component 1 and their weight are shown in Figure 1B. The Venn diagram reveals that 95.5% of the proteins did not significantly differ, and only 4.5% of the proteins were differentially expressed among the MUN groups (*p* > 0,05; Figure 2A). Of the 200 differentially expressed proteins (Supplementary Table 2), we found 80 upregulated and 120 downregulated in the LMU group (Supplementary Table 3). The volcano plot showed a higher expression of IGFBP7 and ND1 in LMU cows, whilst PGAM1, LOC113900017, SLC4A1, CTSC, ERAP1, CHMP5, ERAP1, and EIF4E were less expressed in the LMU group (Figure 2B). Data standardization by Z-score normalization revealed a heat map illustrating the 25 most up- and 25 most downregulated proteins (Supplementary Figure 2).

**FIGURE 1:**
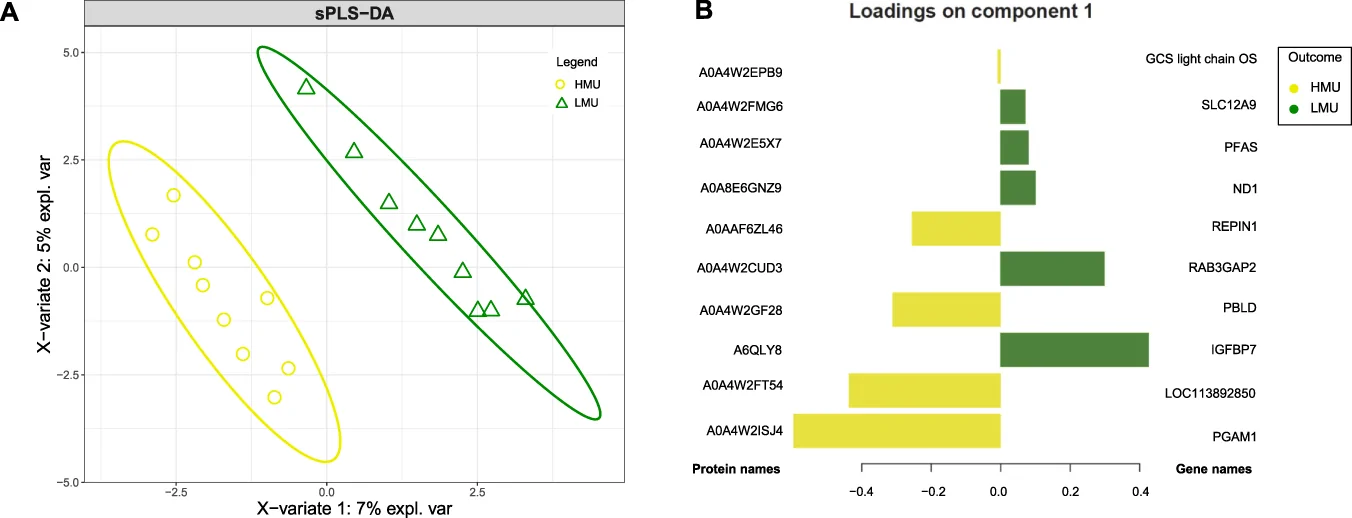
**(A)** Sparse partial least square-discriminant analysis (sPLS-DA) explained 7% variation of component 1 and 5% variation of component 2 of the kidney proteome of lactating dairy cows with low (LMU; green triangles, n = 9) and high (HMU; yellow circles, n = 9) milk urea concentration. **(B)** Loading on component 1 represents calculated loading vector weights of the mean dominating in LMU (green bars) and HMU (yellow bars) cows.

**FIGURE 2:**
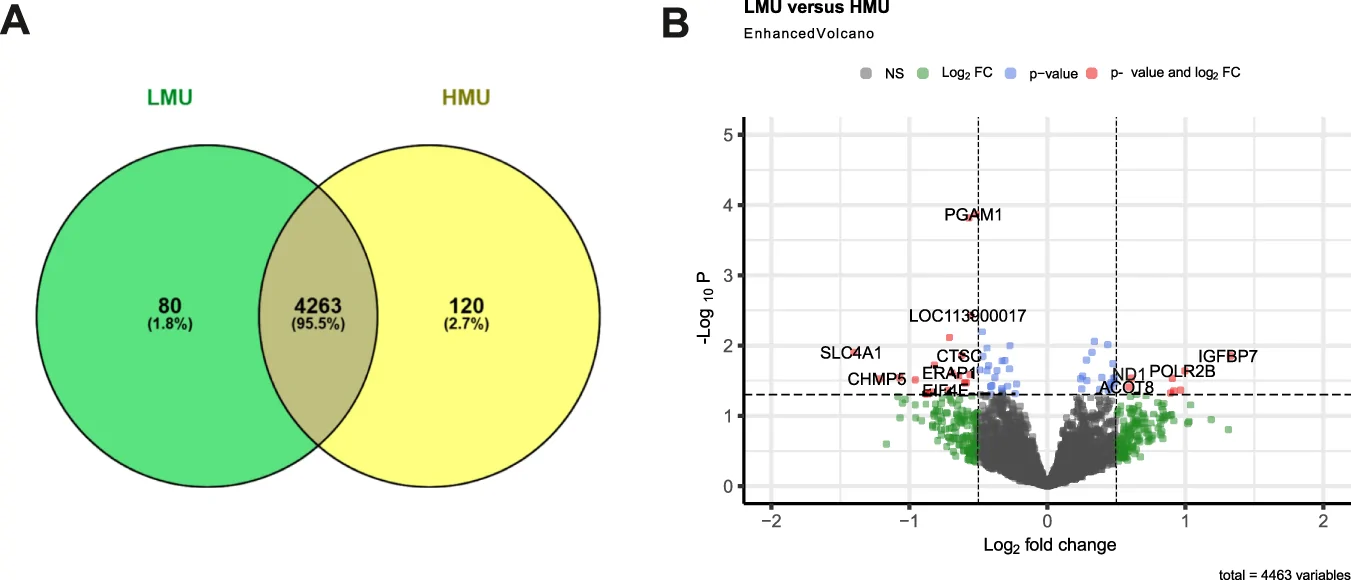
**(A)** Venn diagram showing the number all proteins identified in kidney cortex and medulla tissue. Number and percentage of proteins commonly abundant in both groups (eclipse) and exclusively abundant in dairy cows with low (LMU; green, n = 9) or high (HMU; yellow, n = 9) milk urea concentration is illustrated. **(B)** Volcano plot representing the log_2_ fold-change expression of proteins differentially expressed between LMU vs. HMU groups at −log *p* < 0.05. Top 10 up- and downregulated proteins are highlighted.

### 3.5 Functional enrichment analysis

Upregulated proteins in LMU compared to HMU cows were annotated by biological processes GO terms in DAVID; they were mainly related to dicarboxylic acid catabolic processes, threonine-tRNA aminoacylation, endosome transport regulation, glutamine, and long-chain fatty acid metabolic processes (Figure 3A). Among the molecular function GO category, threonine- and aminoacyl-tRNA ligase and fatty acyl-CoA hydrolase were upregulated, while bisphosphoglycerate mutase, histone deacetylase, and intramolecular phosphotransferase were downregulated in LMU cows (Figure 3B). KEGG analysis identified metabolic, thyroid hormone signaling, and aminoacyl-tRNA biosynthesis pathways to be upregulated and proteasome, glycolysis/gluconeogenesis, glycine, serine and threonine metabolism to be downregulated in LMU compared to HMU cows (Figure 3C). Notably, the transporter for neutral amino acids SLC7A8, peptides (SLC15A2), chloride (SLC12A9), choline (SLC44A1), glucose (SLC2A2), lactate (SLC16A7), and organic anions (SLC22A8) were lower or tended to be expressed in HMU than in LMU cows, whilst the anion exchange protein 1 SLC4A1 and the citrate transporter SLC25A1 tended to be more highly expressed in HMU cows (*p* < 0.09, Table 7).

**FIGURE 3:**
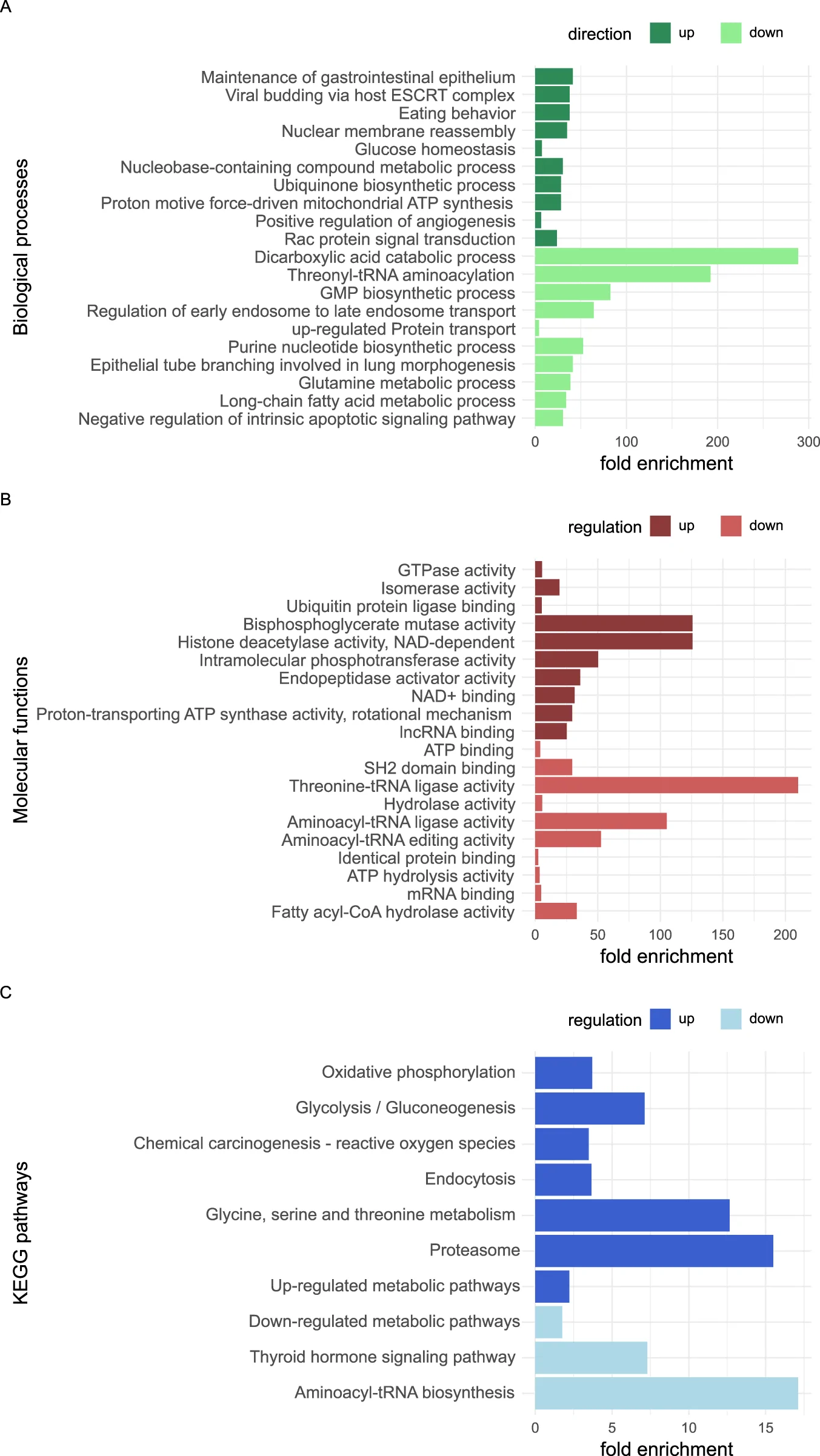
Top 10 GO classifications of proteins based on their involvements in **(A)** biological processes, **(B)** molecular functions, and **(C)** KEGG pathway enrichment analysis for dairy cows with high (HMU, n = 9) and low (LMU, n = 9) milk urea concentration. Data show the up- and downregulated proteins in HMU compared to LMU cows. Fold enrichment was determined as the ratio between the number of target proteins assigned to a specific pathway and the total number of proteins annotated to the pathway in the KEGG database.

**TABLE 7:** Regulation of kidney transporters in HMU compared to LMU cows.

Name	Transporter	Direction	Base mean	*p*-value
SLC7A8	Neutral amino acids	Down	3.68	0.029
SLC22A8	Organic anions	Down	50.84	0.031
SLC12A9	Chloride	Down	4.26	0.053
SLC44A1	Choline	Down	4.97	0.071
SLC2A2	Glucose	Down	7.60	0.072
SLC16A7	Lactate	Down	6.51	0.091
SLC15A2	Peptides	Down	14.88	0.097
SLC4A1	Anion exchange protein	Up	3.64	0.013
SLC25A1	Citrate	Up	30.39	0.066

## 4 Discussion

### 4.1 Kidney weight, renal clearances, and reabsorption

We previously reported that dairy cows with high MUN concentration exhibit elevated plasma urea, creatinine, and uric acid concentrations along with reduced renal clearance rates of these metabolites, suggesting disturbed renal excretion or increased reabsorption by the kidney (Prahl et al., 2023; Prahl et al., 2022). Here, we found that the percentage of total kidney weight in relation to body weight tended to be lower in HMU than in LMU cows, and this trend was mainly due to the significantly lighter right kidney weight relative to body weight. Interestingly, Prahl et al. (2023) also reported lighter right kidney weights in HMU cows fed a low-protein diet but did not normalize the data to body weight. Less kidney parenchyma may account for the reduced urea, creatinine, and uric acid clearance rates they observed earlier. However, the lower kidney weight in HMU cows had no influence on the renal clearance rate for electrolytes (Na^+^ and Cl^−^), lactate, and proteinogenic amino acids, except for isoleucine, for which no concentrations were detectable in urine. Nevertheless, a trend for a lower renal carnosine clearance rate was found in HMU compared to LMU cows, which corresponds to the higher plasma carnosine concentrations, whereas the renal reabsorption rate was not altered in HMU cows. Carnosine, a dipeptide consisting of histidine and beta-alanine, is mainly released from skeletal muscle (Boldyrev et al., 2013) and transported in the kidney by the peptide transporter 2 (PEPT2), also known as “SLC15A2” (Jappar et al., 2009). At increasing arterial plasma carnosine concentrations, the renal clearance of carnosine did not increase, but the kidney cleaves the dipeptide into its monomeric amino acids by carnosinases—at least in dogs (Fukuda et al., 1979). It seems unlikely that the tendency for a lower carnosine clearance rate in HMU cows is due to increased carnosinase activity and is accompanied elevated renal carnosine cleavage because we did not observe the increased plasma histidine concentrations observed in carnosine-infused dogs (Fukuda et al., 1979). Elevated plasma carnosine concentration together with diminished expression of SLC15A2 transporter (PEPT2) (see below) may explain the impaired renal carnosine clearance in HMU cows. Although we did not measure PEPT2 activity in our study, it is known as a high-affinity but low-capacity transporter; its transport rate for carnosine clearance hardly increases when plasma carnosine concentrations increase. Hence, we cannot exclude that the greater carnosine concentration in HMU cows may be caused by an increased release from muscle tissue, but this hypothesis needs testing in future studies. In this case, lower expression of PEPT2 can be considered a counterregulatory response to increasing plasma carnosine concentrations.

In our study, no differences were found in the creatinine clearance rate between HMU and LMU cows, whereas an earlier study reported a greater *RCRcreatinine* for HMU cows (Muller et al., 2021). However, the difference in *RCRcreatinine* between cow groups increased with increasing dietary crude protein concentration (Muller et al., 2021). The 2% lower dietary crude protein concentration fed in the present study may thus explain the different findings for *RCRcreatinine* between studies.

The urea cycle, carried out primarily in the liver and to a lesser extent in the kidney, converts ammonia into urea involving four enzymatic conversion steps and three amino acids: citrulline, arginine, and ornithine. The higher citrulline plasma but comparable ornithine and arginine concentrations suggest reduced urea cycling but greater nitrosative stress in HMU than LMU cows. Increased oxidative stress (see Figure 3C) and reduced expression of genes involved in the urea cycle (see below) of the kidney of HMU cows support this assumption.

We further found a tendency for lower urinary glutamate concentration in HMU cows. Glutamate is not only the carrier of amine groups providing ammonium ions for the synthesis of carbamoyl phosphate entering the urea cycle but also functions as a transporter of ammonia in the glutamate–glutamine cycle at bicarbonate depletion (Wu et al., 2024; Matsumoto et al., 2019). The tendency for lower urinary glutamate concentration together with downregulated glutamine metabolic processes in HMU cows (Figure 3A) suggests less ammonia detoxification by the metabolism of kidney cells in HMU cows. This assumption is again supported by the reduced expression of enzymes related to renal urea synthesis in HMU cows (see below). However, further research is required to elucidate the glutamate–glutamine cycle, renal ammonia/ammonium metabolism, and bicarbonate concentrations in these dairy cows.

### 4.2 Transporters of the kidney proteome

To gain more insight into the kidney metabolism and transport mechanisms, we characterized the renal cortex and medulla proteome of HMU and LMU cows. A variety of kidney transporters were found differently expressed among groups. The lower protein expression of SLC12A9 (potassium: chloride symporter) and SLC22A8 (sodium-dependent transport and excretion of organic anions) in HMU cows might affect their plasma and urine electrolyte concentrations (Hebert et al., 2004) and alter the Na^+^ gradient by organic ion uptake (Tahara et al., 2005). However, we found that none of the corresponding electrolyte concentrations (Na^+^ and Cl^−^) in plasma and urine differed between HMU and LMU cows, and thus no difference in electrolyte clearance and the reabsorption ratio was observed. It has been shown that electrolyte and organic anion transporter expression varies along the nephron (Hebert et al., 2004; Breljak et al., 2016), and thus transporters expressed in the very outer and very inner medulla but not present in the tissue sample analyzed may compensate for the observed expression differences. Furthermore, the increased expression of the ion exchange protein SLC4A1, which exchanges Cl^−^ against bicarbonate, may compensate for the lower SLC12A9 expression and account for comparable Cl^−^ clearance rates among groups.

Similarly, the tendency for a lower protein expression of the lactate transporter SLC16A7 in HMU cows was not associated with different lactate concentrations in plasma or urine. Neither did the respective clearance or reabsorption rates differ between groups. Apart from the inhomogeneous distribution of SLC16A7 in the kidney, other lactate transporters such as monocarboxylate transporters (MCTs) (Wang et al., 2006) affect the efficiency of lactate reabsorption, but those were neither identified in the present study nor found differentially expressed between groups on the RNA level (Reyer et al., 2024). Furthermore, the neutral amino acid transporter SLC7A8 was less expressed in HMU cows, but again no differences in the concentration of a single neutral amino acid in plasma and urine was documented. The regulation of neutral amino acid transporters of the SLC7A family must not be substrate-dependent but rather under the control of acid-base homeostasis (Moret et al., 2007). However, urine pH was comparable between HMU and LMU groups, suggesting potential local pH differences in the kidney. A further reason for the lower SLC7A8 expression but unaltered neutral amino acid reabsorption ratio is that SLC7A8 transports various neutral amino acids, so that changes in the reabsorption of a single neutral amino acid are minor and may be remained undetected. However, the observed differences in SLC transporters may not reflect the number of active transporters in the tubular cell membrane because our proteomic approach involves membrane-bound as well as intracellularly stored, non-functional proteins. Therefore, functional studies are required to confirm our results on total transporter expression.

As mentioned above, we further found a tendency for a lower expression of the peptide SLC15A2 (PEPT2) transporter in HMU cows; it is a proton-coupled peptide transporter for di-, tri- and tetra-peptides, among others, for carnosine (Lichtinger et al., 2024). Although carnosine can also be transported by PEPT1 (SLC15A1), only SLC15A2 was found in less abundance in HMU than LMU cows and linked to higher plasma carnosine concentration in the former. This might explain the tendency for the reduced renal carnosine clearance and greater plasma carnosine concentrations in HMU cows (see above).

Finally, we detected reduced protein abundances of the insulin-independent glucose transporter SLC2A2 (GLUT2) and the choline transporter SLC44A1 and higher expression of the citrate transporter SLC25A1 in HMU cows. This finding was unexpected, and therefore we did not analyze these substrates in plasma and urine. Nevertheless, it remains to be determined in future studies if the altered expression of these three transporters would lead to an altered reabsorption of glucose, choline, and citrate in HMU compared to LMU cows.

### 4.3 Metabolic changes

KEGG pathway enrichment analysis revealed that the thyroid hormone signaling, distinct metabolic pathways, and aminoacyl-tRNA biosynthesis were more downregulated in the kidney cortex and medulla of HMU than LMU cows. Reduced thyroid signaling in the kidney may affect the water and Na^+^ balance and the renin-angiotensin-aldosterone system (RAAS) (Schneider, 1975; Agahi et al., 2024). However, our results provided no further hints for alterations in Na^+^ reabsorption or the RAAS, suggesting the activation of compensatory mechanisms counteracting reduced thyroid signals and maintaining Na^+^ and RAAS homeostasis. Moreover, downregulated metabolic pathways in HMU cows are characterized by lower protein expression of ARG1, a key urea cycle enzyme that converts arginine to ornithine and urea, and proteins related to mitochondrial fatty-acid/branched-chain amino acid oxidation (MCCC2, ACOT4/8). Diminished renal ARG1 activity may reduce local urea formation, which is supposed to affect renal solute handling and the balance between amino acid oxidation and retention, and thus the availability of amino acids for milk protein synthesis (Spek et al., 2012b). Moreover, altered local solute handling may hinder plasma urea clearance and finally increase milk urea concentrations (Spek et al., 2012b).

Lower biosynthesis of aminoacyl-tRNA, which ligates amino acids to their respective transfer RNAs, was mainly caused by the decreased expression of TARS2, YARS1, TARS1, VARS1, and FARS2 proteins in HMU cows. This pathway links translational capacity control with cellular protein synthesis (Reyer et al., 2024). Interestingly, among the genomic regions that influence nitrogen and nitrogen use by the mammary gland of dairy cows, various genes related to aminoacyl tRNA biosynthesis and amino acid transport have been identified (Reyer et al., 2024; Honerlagen et al., 2021; Zhou et al., 2025), but the physiological consequences are still poorly understood and require further investigation.

Among the pathways upregulated in HMU compared to LMU cows were proteasome, endocytosis, and reactive oxygen species. Proteasome is a stress-induced signaling system that degrades misfolded, oxidized, and unused proteins. Upregulation of the proteasomal system is reflected by increased protein expression of 20S alpha subunits (PSMA3/6/7) and 11S regulators (PSME1/2) and is paralleled by the reduced protein synthesis controlled by aminoacyl-tRNAs in HMU cows. Higher protein degradation may be one cause of downregulated protein expression and reduced kidney weight in HMU cows. Specifically, proteasome maintains the renal filtration barrier by degrading renal proteins, thereby preventing them from accumulating at the filtration barrier, which would otherwise reduce filter permeability (Sachs et al., 2024; Delrue and Speeckaert, 2025). Mechanistically, proteasome activates endocytic activity of podocytes, causing increased membrane recycling and endocytic receptor turnover (Sachs et al., 2024; Delrue and Speeckaert, 2025). It can be assumed that the activated proteasome and endocytosis compensate for the limited filtration barrier in low-weight kidneys to maintain filter capacity in HMU cows, but this assumption needs to be proven in future studies. An increased burden may exceed the proteasome’s capacity, causing proteotoxic stress (Sachs et al., 2024; Delrue and Speeckaert, 2025). Whether the increased reactive oxygen signaling in HMU cows is a cause or consequence of increased proteasomal signaling also remains to be examined.

The upregulation of proteasomal, endocytotic, and transport activity requires energy, and this is likely provided by the upregulation of oxidative phosphorylation, glycolysis, and glycine/serine/threonine metabolism. The altered expression of several proteins, such as GAMT, PGAM1, BPGM, PSPH, is linked to the upregulation of the glycine/serine/threonine metabolism. Guanidinoacetate N-methyltransferase (GAMT) converts glycine and arginine to ornithine and guanidinoacetate; the latter is the precursor for the synthesis of creatine/creatine phosphate, a rapidly available energy storage (Stockler et al., 1997). In addition, the increased protein expression of phosphoserine phosphatase (PSPH) may result in increased serine synthesis, the latter facilitating the formation of pyruvate (Pitts and Stone, 1967), which is also the final product of glycolysis.

In this regard, the higher protein expression of phosphoglycerate mutase 1 (PGAM1), bisphosphoglycerate mutase (BPGM), and glucose-6-phosphatase catalytic subunit 1 (G6PC1)) may increase the glycolytic rate (Hallows et al., 2012; Oslund et al., 2017), also providing more pyruvate as an energy substrate for the tricarboxylic acid (TCA) cycle. Interestingly, various renal genes involved in the TCA cycle were found at least numerically upregulated on the mRNA level in HMU compared to LMU cows (Reyer et al., 2024). The TCA cycle provides NADH for mitochondrial ATP production. Greater provision of renal ATP in HMU cows can be directly deduced from the upregulation of oxidative phosphorylation involving the proteins ATP5F1B, NDUFS7, ATP5F1C, and SDHB. These results suggest that increased ATP synthesis facilitates proteasomal, endocytotic, and transport mechanisms in the kidneys of HMU cows.

The present study provides further insights into the molecular regulation of renal clearance in dairy cows with divergent milk urea concentrations. One limitation of our study is that our proteomic approach only assesses total protein abundance rather than membrane localization or transporter, enzyme, and proteasomal activities. Thus, histological and functional studies are required to confirm our mechanistic conclusions drawn from expression analysis. A further drawback is the limited number of animals per milk urea concentration group. The low number may bear the risk of Type II errors and low statistical power and have prevented the inclusion of multiple testing corrections in the statistical model. Therefore, future studies should consider larger populations to validate the results and to draw more generalizable conclusions.

In summary, Holstein dairy cows with high and low milk urea concentrations did not differ in the clearance rate or renal reabsorption ratio for Na^+^, Cl^−^, and lactate, but HMU cows revealed reduced renal carnosine clearance, which was linked to diminished expression of the carnosine transporter SLC15A2 and increased carnosine plasma concentrations. The tendency for lower urinary glutamate concentration together with downregulated glutamine and urea metabolism but increased plasma citrulline concentrations suggests less ammonia detoxification by the metabolism of kidney cells in HMU cows. Decreased renal aminoacyl-tRNA biosynthesis and urea cycling but increased proteasome activity, endocytosis, reactive oxygen species, glycine/serine/threonine metabolism, glycolysis, and oxidative phosphorylation indicates diminished protein synthesis but increased energy production required for the proteasomal machinery for degrading proteins—presumably to prevent clogging of the renal filter barrier. Finally, the earlier observed different urea, creatinine, uric acid, or creatine clearance rates between HMU and LMU cows (Prahl et al., 2023; Prahl et al., 2022) could not be attributed to differences in the expression of their respective transporters but are likely due to altered local solute handling and the lighter relative weight of the right kidney.

## Data Availability

The mass spectrometry proteomics data have been deposited to the ProteomeXchange 466 Consortium via the PRIDE partner repository under the accession number PXD073735. It is available at https://www.ebi.ac.uk/pride/archive/projects/PXD073735, accessible using the password: Skxcb6fp4RW5.
