# Supplementary material for: Renal clearance rate, estimated renal reabsorption ratio, and kidney proteome of Holstein dairy cows with divergent milk urea concentrations
Source: Front Mol Biosci. 2026 Oct 27;13:1819426. doi: 10.3389/fmolb.2026.1819426 (PMC13747852; doi:10.3389/fmolb.2026.1819426)
Supplement: Supplementary file 1 [file Supplementaryfile1.pdf]

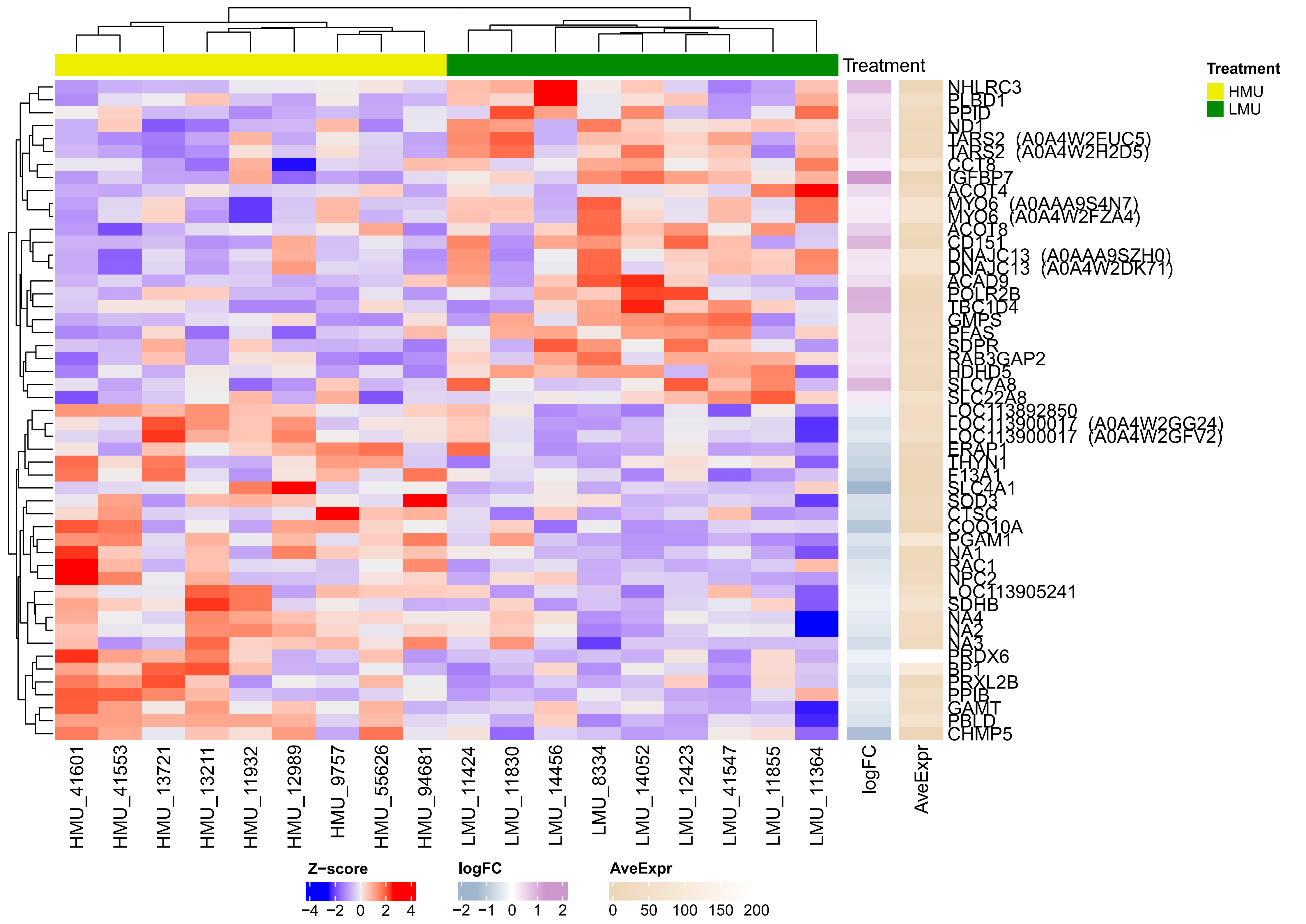
Treatment
Treatment
HMU
LMU
NHLRC3
PLBD1
PPID
ND1
TARS2 (A0A4W2EUC5)
TARS2 (A0A4W2H2D5)
CCT8
IGFBP7
ACOT4
MYO6 (A0AAA9S4N7)
MYO6 (A0A4W2FZA4)
ACOT8
CD151
DNAJC13 (A0AAA9SZH0)
DNAJC13 (A0A4W2DK71)
ACAD9
POLR2B
TBC1D4
GMPS
PFAS
SDPR
RAB3GAP2
HDHD5
SLC7A8
SLC22A8
LOC113892850
LOC113900017 (A0A4W2GG24)
LOC113900017 (A0A4W2GFV2)
ERAP1
THYN1
F13A1
SLC4A1
SOD3
CTSC
COQ10A
PGAM1
NA1
RAC1
NPC2
LOC113905241
SDHB
NA4
NA2
NA3
PRDX6
BP1
PRXL2B
PPIB
GAMT
PBLD
CHMP5
HMU_41601
HMU_41553
HMU_13721
HMU_13211
HMU_11932
HMU_12989
HMU_9757
HMU_55626
HMU_94681
LMU_11424
LMU_11830
LMU_14456
LMU_8334
LMU_14052
LMU_12423
LMU_41547
LMU_11855
LMU_11364
logFC
AveExpr
Z-score
−4 −2 0 2 4
logFC
−2 −1 0 1 2
AveExpr
0 50 100 150 200
